# Task sharing within a managed clinical network to improve child health in Malawi

**DOI:** 10.1186/s12960-015-0053-z

**Published:** 2015-07-21

**Authors:** Bernadette O’Hare, Ajib Phiri, Hans-Joerg Lang, Hanny Friesen, Neil Kennedy, Kondwani Kawaza, Collins E. Jana, George Chirambo, Wakisa Mulwafu, Geert T. Heikens, Mwapatsa Mipando

**Affiliations:** College of Medicine, Blantyre, Malawi; The University of St Andrews, Saint Andrews, UK; Department of Paediatrics and Child Health, College of Medicine, Blantyre, Malawi; Médecins sans Frontières, Allemagne, France; Department of Basic Medical Sciences, College of Medicine, Blantyre, Malawi

**Keywords:** Managed clinical networks, Task shifting, Task sharing, Sub-Saharan Africa, Child health, Paediatrics, Non-physician clinicians

## Abstract

**Background:**

Eighty per cent of Malawi’s 8 million children live in rural areas, and there is an extensive tiered health system infrastructure from village health clinics to district hospitals which refers patients to one of the four central hospitals. The clinics and district hospitals are staffed by nurses, non-physician clinicians and recently qualified doctors. There are 16 paediatric specialists working in two of the four central hospitals which serve the urban population as well as accepting referrals from district hospitals. In order to provide expert paediatric care as close to home as possible, we describe our plan to task share within a managed clinical network and our hypothesis that this will improve paediatric care and child health.

**Presentation of the hypothesis:**

Managed clinical networks have been found to improve equity of care in rural districts and to ensure that the correct care is provided as close to home as possible. A network for paediatric care in Malawi with mentoring of non-physician clinicians based in a district hospital by paediatricians based at the central hospitals will establish and sustain clinical referral pathways in both directions. Ultimately, the plan envisages four managed paediatric clinical networks, each radiating from one of Malawi’s four central hospitals and covering the entire country. This model of task sharing within four hub-and-spoke networks may facilitate wider dissemination of scarce expertise and improve child healthcare in Malawi close to the child’s home.

**Testing the hypothesis:**

Funding has been secured to train sufficient personnel to staff all central and district hospitals in Malawi with teams of paediatric specialists in the central hospitals and specialist non-physician clinicians in each government district hospital. The hypothesis will be tested using a natural experiment model. Data routinely collected by the Ministry of Health will be corroborated at the district. This will include case fatality rates for common childhood illness, perinatal mortality and process indicators. Data from different districts will be compared at baseline and annually until 2020 as the specialists of both cadres take up posts.

**Implications of the hypothesis:**

If a managed clinical network improves child healthcare in Malawi, it may be a potential model for the other countries in sub-Saharan Africa with similar cadres in their healthcare system and face similar challenges in terms of scarcity of specialists.

## Background

### The health system in Malawi

Malawi has a rapidly growing population of currently 16 million, 80% of whom live in rural areas and approximately 50% of whom are less than 15 years old; the paediatrician-to-child ratio is 1:500 000. However, the country has an extensive tiered health system infrastructure based on village clinics, clinics, district hospitals (27 district hospitals and 23 mission hospitals), 4 central hospitals and 2 mental health hospitals [1]. Health facilities are managed by the Ministry of Health (MOH) (60%) or owned and run in collaboration with the Christian Health Association of Malawi (CHAM) (40%). The MOH aims to deliver healthcare services which are within their budgetary and human resource means and aims to provide a basic package of evidence-based care for all Malawians. Constraints to its implementation include weak health system support and shortage of staff [2]. There are also inequities, with the wealthier accessing services more than the poor [3, 4]. The rural poor are particularly at risk for these health inequities [5]. The medical workforce in Malawi consists of non-physician clinicians (medical assistants, clinical officer) and physicians or doctors (medical officers who are generalists and specialists). The former cadres vastly outnumber the latter (see Table 1). Since 1991, doctors have been trained at one institution, the College of Medicine (COM), and 500 medical doctors have graduated. An analysis of recent graduates between 2006 and 2012 showed a retention of 80% but with significant decay each year in the number of graduates still working in the public system (around 50% after 5 years) and less than one third working in rural areas, even shortly after graduation [6]. This is despite the allocation of new graduates to district hospitals [7]. Paediatric specialists working in the public health system in Malawi are located in two of the four central hospitals in Blantyre and Lilongwe. There are no specialists in two referral hospitals or in any of the district or mission hospitals. Since 2004, the COM has been training specialists in paediatrics and child health. This cadre receives 2 years training in Malawi and 2 years in South Africa. Upon passing their professional examinations, these specialists generally take up consultant positions in one of the four central hospitals.

**Table 1:** **Location of paediatric specialists and clinical officers employed by the MOH**

	**2012/13**			
	**Physician clinician**		**Non-physician clinician**	
	**MMED PCH**	**MO**	**CO**	**MA**
QEC Hospital	9			
Blantyre district		36	181	515
Mwanza district		5	143	81
Neno district		14	210	242
Chikhwawa district		2	301	402
Chiradzulu district		10	180	256
Thyolo district		49	208	473
Mulanje district		44	385	423
Phalombe district		12	173	212
Nsanje district		30	202	264
Kamuzu Central	7			
Lilongwe district		147	758	934
Dedza district		21	338	340
Salima district		10	219	505
Mchinji district		10	164	352
Nkhotakota district		22	173	325
Nchisi district		22	195	335
Dowa district		23	329	370
Kasungu district		22	407	374
Zomba Central Hospital	0			
Zomba district		49	326	473
Balaka district		23	184	244
Machinga district		23	130	352
Ntcheu district		21	155	430
Mangochi district		38	250	532
Mzuzu Central Hospital	0			
Mzimba district		70	619	685
Chitipa district		9	183	229
Karonga district		10	206	346
Nkhatabay district		18	221	274

MMED: physician specialists in paediatrics in child health; MO: medical officer, for example, doctors who are not specialists; CO: clinical officers; MA: medical assistant.

*Source*: Health Management Information System (HMIS 2013) Ministry of Health, Malawi.

Malawi, like 25 other countries in sub-Saharan Africa, relies heavily on a cadre who provide diagnostic and clinical services similar to doctors but who are not graduates; they are often described as non-physician clinicians [8]. Malawi has two groups of non-physician clinicians, called clinical officers (COs) and medical assistants (MAs). COs undergo 3 years of training, are awarded a diploma in clinical medicine and serve an internship of 1 year. They offer a broad range of expertise in clinical services, including assessing and treating a wide range of paediatric and adult medical conditions, basic surgery and caesarean sections. MAs receive 2 years training, do not serve internship and do not perform surgery [9]. COs and MAs provide front line medical services in all specialities and at all level of facilities. However, there is no clear career pathway for them to follow upon graduation.

### Managed clinical networks

Managed clinical networks (MCNs) are hierarchically linked groups of professionals and organizations, from primary, secondary and tertiary care, working together across professions and ranks to ensure equitable provision of high-quality healthcare [10]. Successful development depends on the relationships which develop between the professionals and organizations and ideally results in the optimal use of clinicians’ time at each rank. The Royal College of Paediatrics and Child Health has articulated that MCNs have been shown to ensure that the right care is provided as close to home as possible, and clinical networks are fundamental to this aim [11]. A network may be focused on a specific disease or a specialty, and the principles of networking services have been applied in diabetes care, paediatrics, cardiology and vascular surgery. One paediatric model described strives to ensure the right balance between local and specialized services for 400 000 children, and the successes include the use of common standards for management hosted on a website which serves all professionals and the joint ownership of clinical problems across secondary and tertiary providers [12]. The MCN model has also been used in Malawi in the context of the ear, nose and throat (ENT) service. There is one ENT surgeon in the central hospital in the southern region, who has trained 15 clinical officers in ENT, each placed in a district hospital. This has resulted in bringing ENT expertise much closer to the patient with the opportunity for the ENT CO in the district to discuss cases with the ENT surgeon and refer as appropriate [13]. The expected outcomes of MCNs are improved access for those living rurally, equity and quality of care and transfer of knowledge between health professionals with the emphasis shifting from buildings and organizations to services and patients supported by regular meetings and communication between clinicians, shared protocols and guidelines, access to mentoring and regular audit [14]. Given the largely rural population and the scarcity of specialists in rural districts, it is plausible that a managed clinical network model may confer advantages. We hypothesize that paediatricians sharing their expertise within the model of a managed clinical network may improve child health in Malawi.

## Hypothesis

A team of paediatric specialists in all four central hospitals, working within a MCN, and networking with each district hospital in their catchment area through a CO with training in paediatrics will improve healthcare for children in Malawi.

## Testing the hypothesis

To address the problem of insufficient specialists to serve the rural population, the College of Medicine in partnership with the Ministry of Health introduced a specialist Bachelor of Science (BSc) Degree in Paediatric and Child Health (PCH) for COs (and in five other specialties). Experienced COs undertake a 3-year degree in a specific field but retain their abilities to practise in all specialties, for example, the ability to carry out caesarian sections out of hours. The Ministry of Health will consider moving the graduates up on the civil service salary scale which should increase the chances of retention in clinical service; this, combined with a 3-year bond which some of the donors have put in place, should reduce the leakage of a valuable cadre.

The College of Medicine has secured funding for scholarships and additional support to train 33 Bachelor of Sciences for Clinical Officers (BSc CO) in PCH and 16 Masters of Medicine (MMED) paediatric specialists by 2020, (see “Acknowledgements” section for partners). In addition to the current establishment, there will be sufficient MMED specialist paediatricians to staff all four central hospitals. The 33 BSc CO in PCH are sufficient to staff each government district hospital with this new cadre. The training of the BSc CO PCH includes one semester of basic medical sciences, two semesters where they are supervised in a district hospital and three semesters in the central hospital. During their first district semester (the junior clinical attachment), they concentrate on acute paediatrics in the district hospital. In the second district semester (the senior clinical attachment), the focus will be on skills which will allow them to function optimally as district leaders for children. This will include effective collaboration with existing child health services and skills in advocacy in order to improve the determinants of health, such as water and sanitation at a local level. Post-graduation, for 1 year, they continue to receive mentorship from the specialist paediatricians working in the central hospital to which they refer. The vision is that with mentorship, they will become leaders in their district not only in acute paediatric care but also by engaging with existing preventative health services, acting as custodians of World Health Organization (WHO) guidelines and advocating on issues pertaining to the determinants of health. As a result of their undergraduate training and postgraduate mentoring, delivered by their paediatric counterparts based in central hospitals, a robust network will become embedded, with the specialists in the central hospital sharing the responsibility within their network of CO colleagues in the district. Other benefits will include the timely cascade of updated guidelines, email and phone communication and avoidance of unnecessary travel by patients. The logistics of the MCN, including standard operating policies, funding for mentoring visits and on-line support in the form of a virtual learning environment is in place. In order to support the integration of the MCN with existing services, programme managers at the Ministry of Health and the District Health Management Team (DHMT) will be engaged to arrive at a mutual understanding of roles.

We will use a natural experiment model and compare annually over the next 5 years; data from all districts will be collected at baseline and annually until 2020 as the specialists of both cadres take up posts. A comparison of data for the period before and after, as well as between the districts will be done. Districts will be analysed as posts are filled according to which category they fall that year. For example, if a district has a BSc CO PCH in post and is being mentored by specialists from the referral hospital, the data from this district will be analysed as being in arm 4 for that year (see Table 2). In 2016, with the return of the first cohort to its own district, we will begin to evaluate the impact of the managed clinical network. Prior to collecting and analysing data, we will have submitted a full proposal and received approval from the College of Medicine Research and Ethics Committee (COMREC) [15]. This is an independent scientific and ethics committee and is responsible to the College of Medicine and to the Government of Malawi.

**Table 2:** **Potential combination of specialist cover in each district**

**Arms**		**MMED specialist**	**BSc PCH CO**
1	Control arm	X	X
2	BSc CO arm	X	√
3	MMED ARM	√	X
4	MCN	√	√

MMED: masters in medicine in paediatrics; BSc: Bachelors of Science; PCH: paediatrics and child health; CO: clinical officer; MCN: managed clinical network.

The MOH routinely collects data using a Health Management Information System (HMIS) which includes 128 indicators, of which many are relevant to children. We will collect data on the case fatality rate for the common causes of under-5 mortality and process indicators for the quality of care provided using the World Health Organization integrated maternal, neonatal and child quality of care assessment and improvement tool. Limitations of the routinely collected data quality will be addressed by corroborating with data collected by the clinician locally.

## Implications of the hypothesis

If a managed clinical network, whereby specialists in central hospitals are closely linked to non-physician specialists in district hospitals reduces case fatality rates and improves quality of care process indicators and thus equity of care between rural and urban, it may be a potential model for other countries in the region which face similar challenges to consider.

## Abbreviations

BSc CO: Bachelor of Sciences for Clinical Officers
CHAM: Christian Health Association of Malawi
CO: Clinical officers
COM: College of Medicine
COMREC: College of Medicine Research and Ethics Committee
DHMT: District Health Management Team
ENT: Ear, Nose and Throat
HMIS: Health Management Information System
MA: Medical assistant
MCN: Managed clinical networks
MMED: Masters of Medicine
MOH: Ministry of Health
PCH: Paediatrics and Child Health
WHO: World Health Organization
